# *Prosapia bicincta* (Hemiptera: Cercopidae) abundance, plant associations, and impacts on groundcover in Hawaiʻi Island rangelands

**DOI:** 10.1093/ee/nvae062

**Published:** 2024-07-02

**Authors:** Shannon Wilson, Mark S Thorne, Melissa A Johnson, Daniel C Peck, Mark G Wright

**Affiliations:** Department of Plant and Environmental Protection Sciences, University of Hawaiʻi at Mānoa, Honolulu, HI, USA; Daniel K. Inouye US Pacific Basin Agricultural Research Center, United States Department of Agriculture–Agricultural Research Service, Hilo, HI, USA; Oak Ridge Institute for Science and Education, United States Department of Energy, Oak Ridge, TN, USA; Department of Human Nutrition, Food and Animal Sciences, University of Hawaiʻi at Mānoa, Kamuela Cooperative Extension Office, Kamuela, HI, USA; Daniel K. Inouye US Pacific Basin Agricultural Research Center, United States Department of Agriculture–Agricultural Research Service, Hilo, HI, USA; Vestaron Corp, Field Development, Kalamazoo, MI, USA; Department of Plant and Environmental Protection Sciences, University of Hawaiʻi at Mānoa, Honolulu, HI, USA

**Keywords:** invasive species, forage pest, range management, tropical pasture, Integrated Pest Management

## Abstract

The twolined spittlebug, *Prosapia bicincta* (Say), is a major economic pest of forage grass and turfgrass. *Prosapia bicincta* was first detected in rangelands on Hawaiʻi Island in 2016 and has since spread to an estimated 72,000 ha in the North and South Kona districts. This study aimed to quantify *P. bicincta* abundance, plant associations, and impacts on groundcover over time. Monthly surveys of *P. bicincta* nymphs and adults were conducted from February 2018 to September 2022 along 17 established 100-m transects at 4 ranches located in Kona, Hawaiʻi Island, spanning an elevation gradient from 519 to 1,874 m above sea level (a.s.l.). Monitoring revealed *P. bicincta* occurs from 519 to 1,679 m a.s.l., primarily in Kikuyu grass (*Cenchrus clandestinus* (Hochst. ex Chiov.)) Morrone (Poales: Poaceae) pastures. Peaks in *P. bicincta* abundance coincided with the wet season, with most activity occurring from April to October and little to no activity between November and March. Mid elevation (1,000–1,300 m) transects had significantly higher mean *P. bicincta* abundance (126 nymphs/m^2^) relative to low (500–999 m) (64 nymphs/m^2^) and high elevations (>1,300 m) (20 nymphs/m^2^). Sites with the highest abundance of *P. bicincta* were also associated with the greatest decrease in mean grass cover (30%) and were replaced by forbs, bare ground, and shrubs. Grasses accounted for 72% of the total *P. bicincta* detections, with the remaining plants comprised of legumes (16%), sedges (6%), and forbs (6%). Twenty new *P. bicincta* plant associations were found. This information will help improve the effectiveness of management to suppress populations below economic thresholds.

## Introduction

*Prosapia bicincta* (Say), or the twolined spittlebug, is a pasture and turfgrass pest native to the southeastern United States where it is distributed from Florida to Maine and as far west as Texas (Shortman et al. 2002, Thompson and Carvalho 2016). *Prosapia bicincta* completes 2–3 generations per year depending on the region (Pass and Reed 1965, Fagan and Kuitert 1969, Buss and Williams 2011). *Prosapia bicincta* negatively impacts rangelands by feeding on forage grasses such as coastal Bermuda (cultivar of Cynodon dactylon (L.) Pers.) and pangola (*Digitaria eriantha* Steud.) (Byers and Wells 1966, Shortman et al. 2002). In late 2016, *P. bicincta* was detected in Kainaliu in the South Kona district of Hawaiʻi Island (Wilson et al. 2023), making it the first species of Cercopidae to invade the state. From 2017 to 2020, *P. bicincta* rapidly expanded its range at a rate of over 14,000 ha per year (Wilson et al. 2023). Though the rate appeared to slow by 2020, *P. bicincta* was already occupying an estimated 72,183 ha in Kona by 2021 (Wilson et al. 2023).

The beef cattle industry is economically, ecologically, and culturally significant in Hawaiʻi. Cattle first arrived in the islands in the late 1700s (Starrs 2000, Barna 2013, Mills et al. 2013). By the early 1800s, paniolos (Hawaiian cowboys) developed a unique style of ranching that incorporated their culture and knowledge of the land (Barna 2013, Mills et al. 2013). Multicultural immigrants joined the ranching community in the following decades, and by the early 1900s cattle ranching was a well-established industry (Barna 2013, Mills et al. 2013, Melrose et al. 2016). Pasture lands currently make up nearly 20% of Hawaiʻi’s land area and when well-managed, pastures provide several ecosystem services including clean water, soil retention, soil carbon storage, improved soil health, flood reduction, suppression of invasive weeds, open spaces for recreation, and reduction of fire risk (Bremer et al. 2021). Beef cattle are the third largest agricultural commodity in Hawaiʻi with an estimated value of $48 million annually (HDOA and USDA-NASS 2022).

Kikuyu (*Cenchrus clandestinus* (Hochst. ex Chiov.) Morrone) and pangola (*Digitaria eriantha* Steud) are key livestock forage grasses that support nearly 70% of Hawaiʻi’s cattle and have sustained ranching for several decades (Asem-Hiablie et al. 2018). Kikuyu grass is the most widespread and important forage in Hawaiʻi but is extremely susceptible to *P. bicincta* feeding. Thus, *P. bicincta* is considered a severe threat to thousands of hectares of Kikuyu grass pastures and to the sustainability of the forage-based beef cattle industry in Hawaiʻi.

In the present study, we quantified the seasonal abundance and distribution of *P. bicincta* over nearly 5 yr on Hawaiʻi Island and examined plant associations and impacts on vegetative cover within pastures. The information on seasonality will elucidate *P. bicincta* developmental rates under local conditions, which will help determine when to employ management tactics to target critical points in the lifecycle and suppress populations below economic thresholds.

## Methods

### Field Surveys

**Fig. 1.**
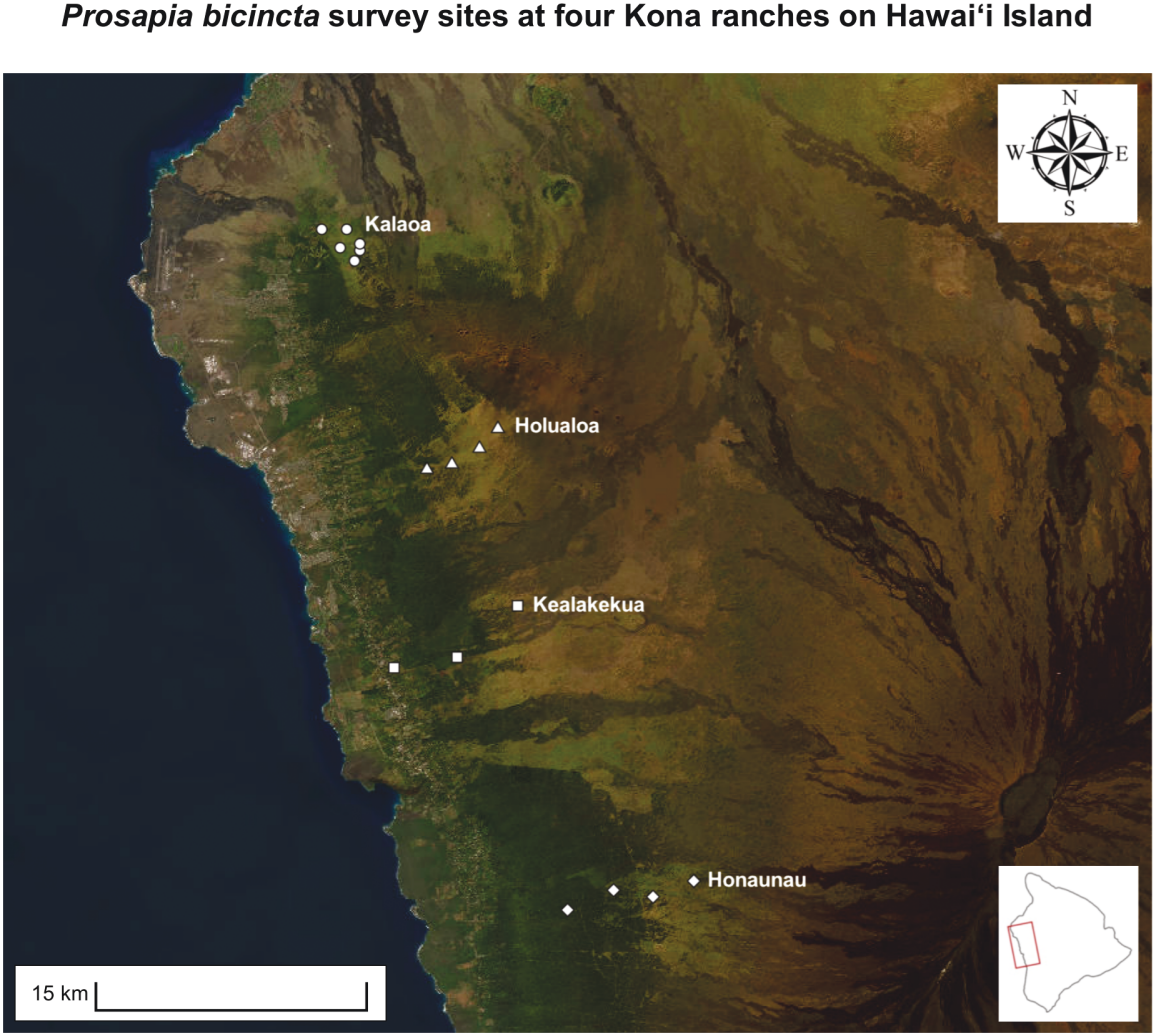
Map of geographic distribution of 4 ranches and 17 transects surveyed for *Prosapia bicincta* in North and South Kona districts, west Hawaiʻi Island. Transect elevations ranged from 736 to 1,049 m at Kalaoa (circles), 1,072 to 1,605 m at Holualoa (triangles), 519 to 1,194 m at Kealakekua (squares), and 1,102 to 1,874 m at Honaunau (diamonds). Inset map shows the area surveyed on Hawaiʻi Island.

Based on initial reports of *P. bicincta* in the North and South Kona districts of Hawaiʻi Island, study sites were selected along an elevational gradient from **~** 600 to 1,500 m above sea level (a.s.l.), and in surrounding areas that were identified as high-risk for infestation (see Wilson et al. 2023). The 4 ranches used in this study were in Kalaoa, Holualoa, Kealakekua, and Honaunau (Fig. 1). The northernmost ranch was located on the west-facing slopes of Huālalai and the southernmost ranch on the west-facing slopes of Mauna Loa. Three to six 100-m transects were permanently established at each ranch, with a total of 17 transects across the 4 ranches: 6 at Kalaoa, 4 at Holualoa, 3 at Kealakekua, and 4 at Honaunau. These transects encompassed a range of elevations from 519 to 1,874 m a.s.l. and were monitored monthly from February 2018 to September 2022. Starting from the lowest elevation on each ranch, transects were placed in parallel to each other but separated by approximately 300- to 450-m increments in altitude to span the full elevation gradient encompassed by each ranch. Exact transect placement was influenced by habitat suitability at each elevation.

The elevation of each transect varied across ranches since each ranch encompassed a range of different elevations. The starting point of each transect was randomly selected and traversed upslope along the elevational gradient. Each transect was delineated using a 100-m tape. To sample nymphs, a circular 0.25-m^2^ quadrat was placed every 10 m along each transect, alternating left and right sides and 1 m from the tape. All spittle masses in the ring were inspected to record number of nymphs and plant on which they occurred. This typically required digging through the grass thatch to the root level for grasses. Adult *P. bicincta* abundance was assessed at each transect site using a heavy-duty insect sweep net. At each transect the right or left side of the tape was randomly selected for sampling, with the starting point being 15 m out from the transect line. One adult sweep net sample was taken along the length of the transect, which consisted of sweeping 50 times in a zig zag pattern. After sweeping, the number of adults in the net was counted.

Plant community composition was quantified in each 0.25-m^2^ quadrat by identifying all the plant species present and estimating the percent bare ground and vegetative cover. Plants were first assigned to growth form (grass, forb, or shrub) and groundcover was assessed visually by estimating the percent vegetation cover or soil/bare ground. Plant species were then identified using identification keys for Hawaiʻi grasses (Hitchcock 1922, Whitney et al. 1939) and weeds (Motooka et al. 2003).

### Wet and Dry Season Parameters

Local climate conditions can drastically differ on Hawaiʻi Island due to the trade winds and unique topography, producing a multitude of microclimates that can vary greatly within just a few km (Robyns and Lamb 1939, Giambelluca et al. 2013). The Rainfall Atlas of Hawaiʻi (Giambelluca et al. 2013) was used to define the wet season across field sites using the closest weather stations. The selected stations provided consistent and long-term records ranging from the early 1900s to 2012. The ‘mapped mean’ rainfall values were estimated by weather station rain-gauge data and estimates from rainfall radars, modeling, and analyses (Giambelluca et al. 2013). We defined the wet season as months exceeding 100 mm of rainfall. Based on the mean of all the selected stations, monthly rainfall surpassed 100 mm from April to October and was highest from May to September. Thus, April–October was considered the wet season for our analyses. With rainfall below 100 mm, November–March was considered the dry season.

### Statistical Analysis

Generalized linear mixed models (GLMMs) were used to determine if season and elevation were factors indicative of *P. bicincta* nymph and adult abundance while accounting for random effects (ranch and year). Separate GLMMs were run for the 2 different continuous response variables of interest (nymph and adult abundance). Both GLMMs were run using negative binomial distributions to account for overdispersion. For each GLMM, season and elevation were fixed effects and grouped into categorical variables of wet season months (April–October) or dry season months (November–March), and elevation ranges of low (500–999 m a.s.l.), mid (1,000–1,300 m a.s.l.), or high (> 1,300 m a.s.l.). The elevation ranges were grouped based on observed heterogeneity in habitats and climate conditions. Site (ranch) and year (2018–2022) were defined as categorical variables and included as random effects in the model. GLMMs and model comparisons using likelihood ratio tests were carried out using the R glmer function in the lme4 package (Bates et al. 2015). Pairwise Tukey comparisons were done with the emmeans package (Lenth 2023) in R version 4.3.1 (R Development Core Team 2023).

Plant community data were quantified by mean percent cover of the functional groups (grass, forb, and shrub) or bare ground per transect, and changes were assessed over the 5 yr sampled. All variables were tested for normality by visual inspection of histograms and quantile-quantile plots and by performing a Shapiro–Wilk test. A Wilcoxon–Mann–Whitney test was conducted to compare changes in mean grass cover and determine if grass cover was significantly different in 2018 and 2022 for each elevation group. Analyses were conducted using the stats package in R version 4.3.1 (R Development Core Team 2023).

## Results

### Nymph and Adult Abundance

**Fig. 2.**
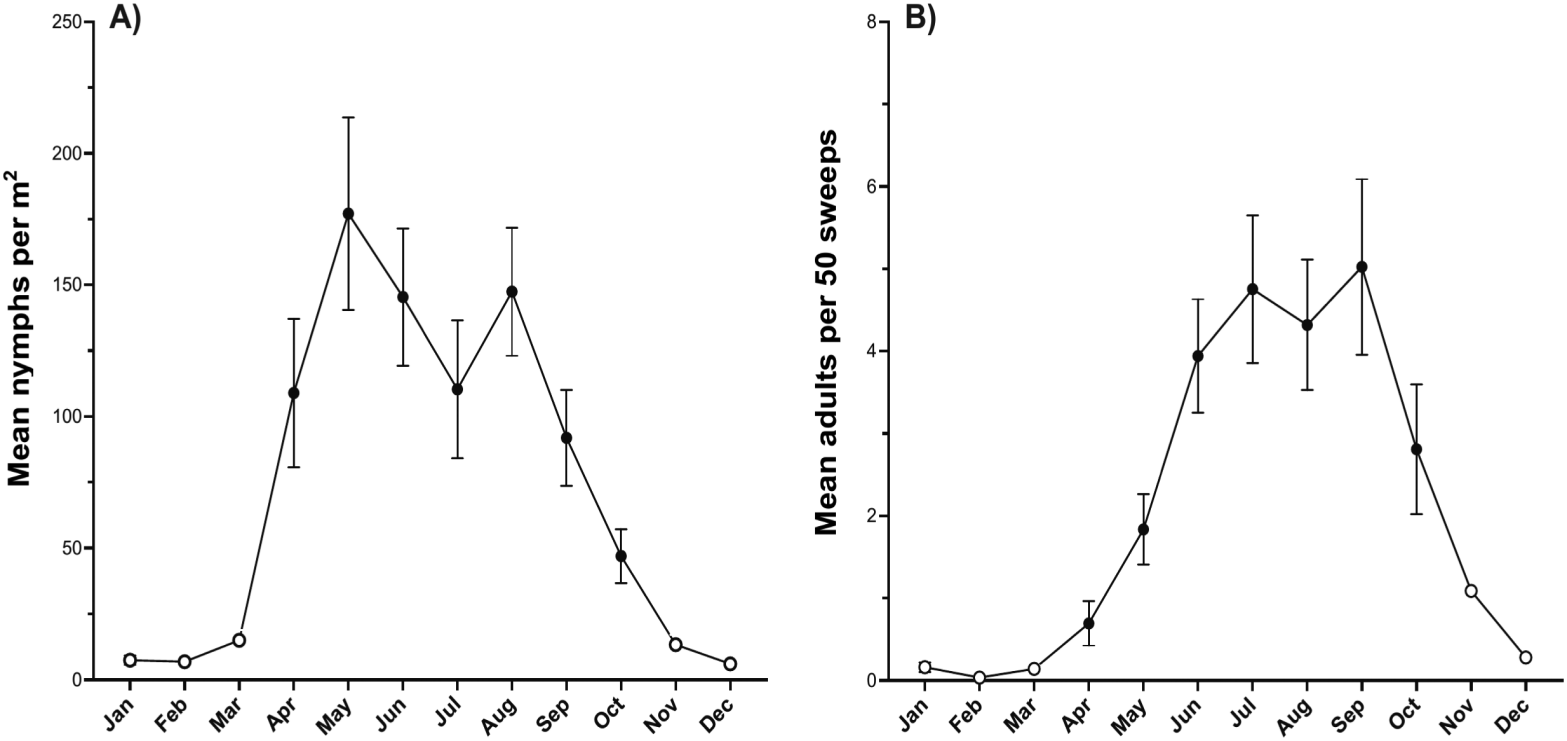
Seasonal variation in *Prosapia bicincta* A) nymph and B) adult abundance (mean ± SEM) in North and South Kona districts, west Hawaiʻi Island, across all years, elevations, and ranches surveyed. Dry season months are shown in white circles, wet season months in black circles.

From February 2018 to September 2022, a total of 73,272 nymphs and 2,058 adults were collected across the 17 transects and 4 ranches. The surveys showed distinct seasonal fluctuations in *P. bicincta* nymph and adult abundance. Results from the GLMM showed that season and elevation were good predictors of both nymph abundance (*X*^2^ = 138.93, *df* = 1, *P* ≤ 0.001) and adult abundance (*X*^2^ = 148.79, *df* = 1, *P* ≤ 0.001) in Kona pastures. Across all sites and years, mean nymph and adult abundance was significantly higher in wet season versus dry season months (Table 1). Increased nymph abundance coincided with the wet season with most activity occurring between April and October, with little to no activity between November and March. Nymph abundance rapidly increased in April at the start of the rainy season and peaked in May (Fig. 2A). A second peak was observed in August, after which abundance steadily declined through September and October. Fewer nymphs were detected once the dry season returned in November. Of the total nymphs sampled, 95% were detected during the wet season and 5% during the dry season. Adult abundance reflected patterns observed in nymph abundance with 2 peaks and most activity occurring during the wet season. Adult abundance was highest from May to November and lowest from December to April, with peaks occurring in July and September (Fig. 2B). Peak adult abundance occurred about 1–2 months after peaks in nymph abundance. Of the total adults sampled, 94% were detected during the wet season and 6% during the dry season.

**Table 1.** Generalized linear mixed model post-hoc comparisons of explanatory variables (season, elevation) on *Prosapia bicincta* nymph and adult abundance

	Tukey contrasts			
Model variable	Estimate	SE	z-ratio	*P*-value
*Nymph abundance*				
Dry season months vs wet season months	−2.35	0.17	−13.78	<0.0001
Low elevation vs mid elevation	−0.57	0.21	−2.71	0.0184
High elevation vs low elevation	−1.11	0.30	−3.65	0.0008
High elevation vs mid elevation	−1.68	0.23	−7.44	<0.0001
*Adult abundance*				
Dry season months vs wet season months	−2.29	0.17	−13.42	<0.0001
Low elevation vs mid elevation	−0.39	0.19	−2.04	0.1027
High elevation vs low elevation	−1.40	0.29	−4.84	<0.0001
High elevation vs mid elevation	−1.80	0.22	−8.24	<0.0001

**Fig. 3.**
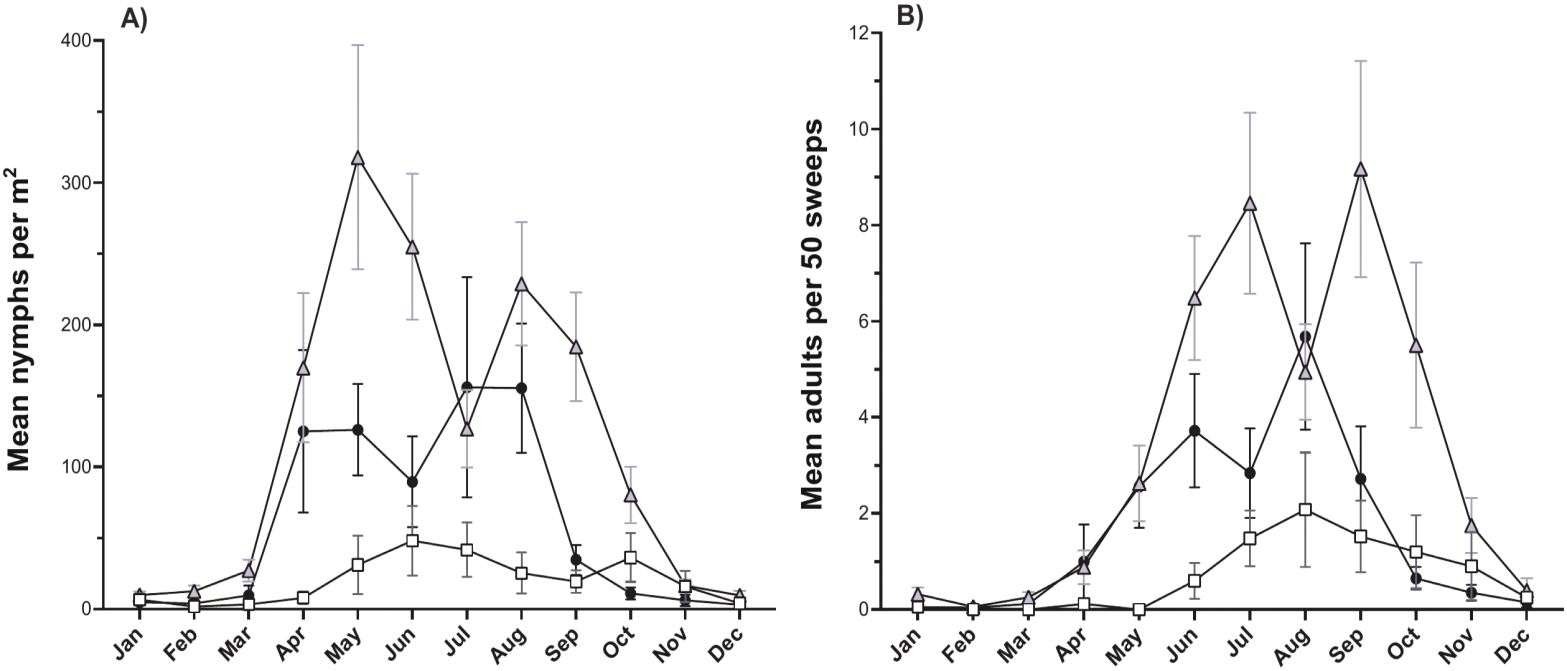
Seasonal variation in *Prosapia bicincta* A) nymph and B) adult abundance (mean ± SEM) by elevation. Low elevation = 500–999 m (black circles), mid elevation = 1,000–1,300 m (gray triangles), and high elevation > 1,300 m (white squares).

There were significant differences in the abundance and timing of nymphs across elevations (Table 1; Fig. 3A). Of the total nymphs sampled, 68% were at mid elevations, 25% at low-elevations, and 8% at high-elevations. At mid elevations, abundance was highest from April to October, with peaks in May and August (Fig. 3A). Nymph abundance in the low elevation also increased rapidly in April with peaks in May and August, but decreased by September, a month earlier than the mid elevation (Fig. 3A). Seasonal nymph abundance was much lower at high elevations and activity occurred later (Fig. 3A). Most activity at high elevation was from May to November with peaks in June and October. No nymphs were detected at 1,874 m and very little activity was detected above 1,605 m.

Patterns of adult abundance by elevation paralleled those observed in nymphs (Table 1). Adult abundance was highest at mid elevations (Fig. 3B). Most adult activity at mid elevation occurred from May to November, with peaks in June and September. At low elevations, adults were mainly detected from May to September and peaks occurred in June and August (Fig. 3B). Adult abundance at high elevation was much lower than at mid and low elevations, with less fluctuation throughout the season and more gradual increases and decreases in abundance. At high elevations, adult abundance was highest from June to November and peaked in August. No adults were detected above 1,679 m and very little adult activity was detected at the 1,427 and 1,605 m transects. There were 2 peaks in adult abundance in the low- and mid-elevation groups while high-elevations had only one (Fig. 3B).

Nymph and adult abundance increased during the first 3 yr of the study and then declined in each of the last 2 yr (Supplementary Fig. S1; Supplementary Fig. S2). Of the total nymphs sampled, 77% were observed from 2018 to 2020, 17% in 2021, and only 6% in 2022 (Table 2). The maximum density of 2,352 nymphs/m^2^ was observed in 2018 (Table 2). Similarly, of the total adults sampled, 73% were observed from 2018 to 2020, 18% in 2021, and 10% in 2022 (Table 2). The maximum count of 73 adults per 50 sweeps was observed in 2020.

**Table 2.** Annual *Prosapia bicincta* nymph densities (per m^2^) and adult counts (per 50 sweeps) across all ranches surveyed in North and South Kona districts, west Hawaiʻi Island. Showing total number of surveys conducted (*N*), mean density or count, max density or count, and total abundance of nymphs and adults detected each year

		Nymphs (per m^2^)			Adults (per 50 sweeps)		
Year	N	Mean density	Max density	Total abundance	Mean count	Max count	Total abundance
2018	187	92	2,352	17,264	2	21	325
2019	204	90	1,856	18,280	2	49	489
2020	204	103	1,160	20,956	3	73	669
2021	204	60	980	12,180	2	35	373
2022	153	30	408	4,592	1	22	202

Lastly, Kealakekua had the highest mean density of nymphs (133 nymphs/m^2^) followed by Holualoa (107 nymphs/m^2^), Kalaoa (60 nymphs/m^2^), and Honaunau (31 nymphs/m^2^) (Table 3; Supplementary Fig. S3). The mean number of adults per sweep net sample was highest at Holualoa (4 adults/50 sweeps) followed by Kealakekua (3 adults/50 sweeps), Kalaoa (2 adults/50 sweeps), and Honaunau (1 adult/50 sweeps) (Table 3; Supplementary Fig. S4). At each ranch, seasonal patterns in nymph (Supplementary Fig. S5) and adult (Supplementary Fig. S6) abundance varied by elevation.

**Table 3.** Geographic distribution of *Prosapia bicincta* nymph densities (per m^2^) and adult counts (per 50 sweeps) across all years of surveys in Kona, west Hawaiʻi Island. Showing the 4 ranches surveyed by their location within North and South Kona districts, total number of transects established at each ranch, elevation range (m a.s.l.), total surveys conducted/number of transects sampled (*N*), mean density or count, max density or count, and total abundance of nymphs and adults detected

			Nymphs (per m^2^)			Adults (per 50 sweeps)		
Ranch (# transects)	Elevation (m a.s.l.)	N	Mean density	Max density	Total abundance	Mean count	Max count	Total abundance
Kalaoa (6)	736–1,049	336	60	1,284	20,000	2	49	516
Holualoa (4)	1,072–1,605	224	107	1,008	23,968	4	73	899
Kealakekua (3)	519–1,194	168	133	2,352	22,284	3	30	461
Honaunau (4)	1,102–1,874	224	31	1,160	7,020	1	23	182

### Plant Associations

The spittle masses of *P. bicincta* nymphs were detected on 32 different plants in the Kona pastures surveyed (Table 4). Grasses accounted for 72% of the total associations, with the remaining plants comprised of legumes (16%), sedges (6%), and forbs (6%). We report 20 new associations of plants utilized by *P. bicincta* nymphs: 13 grass species, 4 legume species, 2 forb species, and 1 sedge species (Table 4). It is uncertain if nymphs completed their life cycle on these plants, but the presence of spittle containing nymphs was observed.

**Table 4.** Plant associations for *Prosapia bicincta* nymphs in rangelands located in North and South Kona districts, west Hawaiʻi Island. Nymphs were detected on 23 different grass taxa, 5 legume species, 2 forb species, and 2–3 sedge taxa

Family	Scientific name	Common name
Asteraceae	*Erigeron canadensis* L.	Marestail^a^
Asteraceae	*Senecio madagascariensis* Poir.	Fireweed^a^
Cyperaceae	*Cyperus* L. spp. (2–3 taxa)	Cyperus sedge
Cyperaceae	*Cyperus brevifolius* (Rottb.) Hassk.	Kyllinga sedge^a^
Fabaceae	*Crotalaria* L. spp.	Rattlepod^a^
Fabaceae	*Desmodium incanum* DC.	Kaʻimi clover^a^
Fabaceae	*Glycine wightii* (Wight and Arnott) Verdc	Glycine^a^
Fabaceae	*Lotus corniculatus* L.	Birdsfoot trefoil^a^
Fabaceae	*Trifolium repens* L.	White clover
Poaceae	*Anthoxanthum odoratum* L.	Sweet vernal^a^
Poaceae	*Axonopus fissifolius* (Raddi) Kuhlm.	Carpetgrass^a^
Poaceae	*Briza minor* L.	Little quaking grass^a^
Poaceae	*Cenchrus clandestinus* (Hochst. ex Chiov.) Morrone	Kikuyu^a^
Poaceae	*C. setaceus* (Forssk.) Morrone.	Fountain^a^
Poaceae	*C. clandestinus *×* C. setaceus* hybrid	Kikuyu-Fountain hybrid^a^
Poaceae	*Cynodon dactylon* (L.) Pers.	Bermuda
Poaceae	*Digitaria* Haller. spp.	Digitaria spp.
Poaceae	*Digitaria eriantha* Steud.	Pangola
Poaceae	*Elymus hystrix* L.	Bottlebrush^a^
Poaceae	*Holcus lanatus* L.	Yorkshire fog^a^
Poaceae	*Megathyrsus maximus* B.K.Simon & S.W.L. Jacobs.	Guinea
Poaceae	*Microlaena stipoides* (Labill.) R.Br.	Meadow rice grass^a^
Poaceae	*Panicum repens* L.	Wainaku grass
Poaceae	*Paspalum* L. spp.	Paspalum spp.
Poaceae	*Paspalum conjugatum* P.J.Bergius.	Hilo grass^a^
Poaceae	*Paspalum notatum* Flüggé.	Bahiagrass
Poaceae	*Sacciolepis indica* (L.) Chase.	Glenwood grass^a^
Poaceae	*Setaria parviflora* (Poir.) Kerguélen.	Foxtail
Poaceae	*Sporobolus indicus* (L.) R. Br.	Rattail^a^
Poaceae	*Stenotaphrum secundatum* (Walter) Kuntze	St. Augustine
Poaceae	*Urochloa* (*Brachiaria*) P. Beauv. spp.	Brachiaria spp.
Poaceae	*Urochloa decumbens* (Stapf) R.D.Webster	Signal^a^

^a^ = new food-plant record for *Prosapia bicincta*.

### Changes in Groundcover

**Fig. 4.**
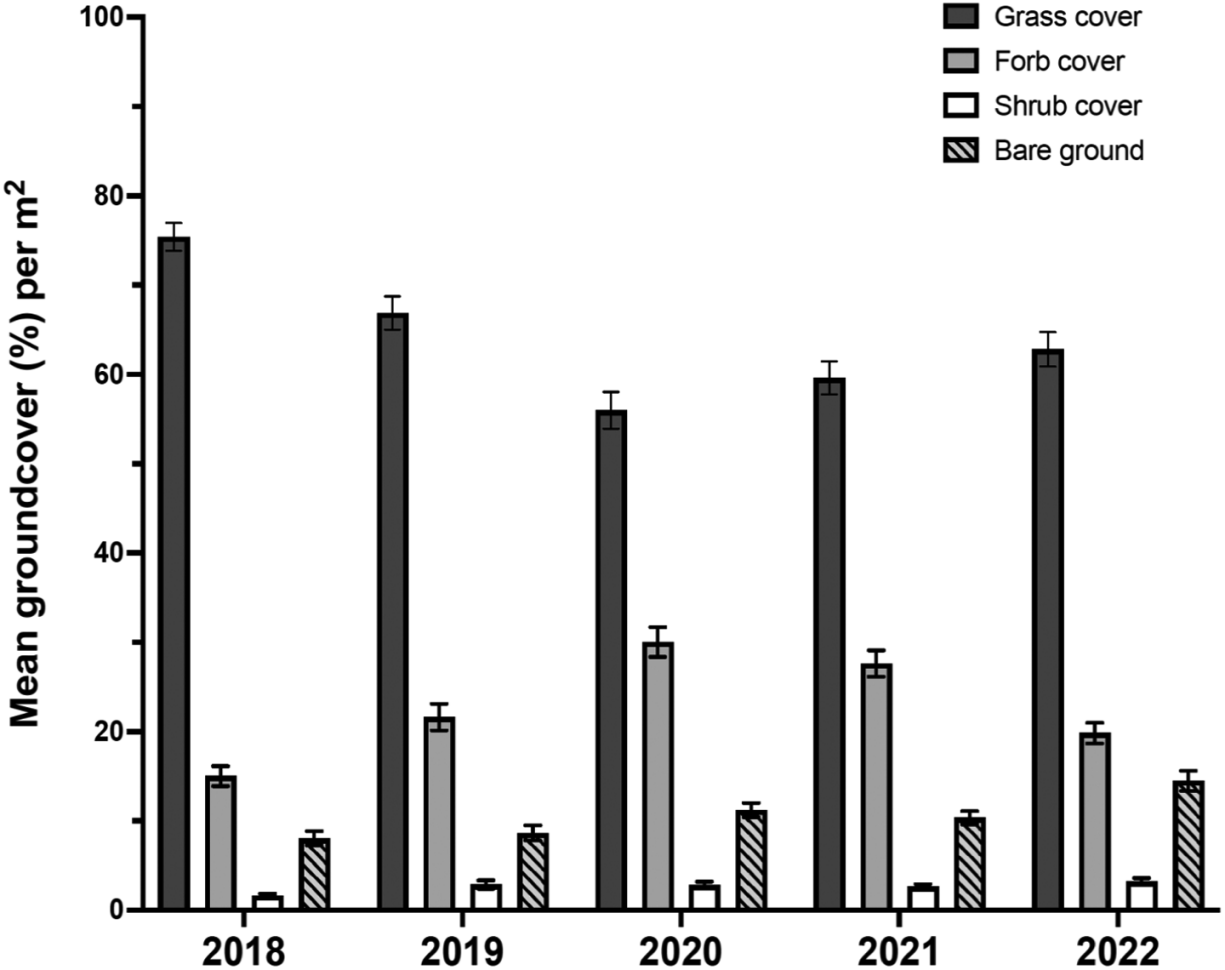
Annual variation in proportion of mean groundcover (±SEM) during 2018–2022 across all 4 ranches in North and South Kona districts, west Hawaiʻi Island.

Mean grass cover was significantly higher in 2018 than in 2022 at low (73% vs. 57%) (Wilcoxon–Mann–Whitney; *W* = 1749, *P* = 0.0004) and mid (68% vs. 47%) (*W* = 3852, *P* < 0.0001) elevations. In contrast, there was no difference in mean grass cover at high elevations (89% in 2018 vs. 90% in 2022) over the 5-year study period (*W* = 1243, *P* = 0.975). As grass cover decreased from 2018 to 2022, forb cover and bare ground increased, and shrub cover remained stable (Fig. 4).

**Fig. 5.**
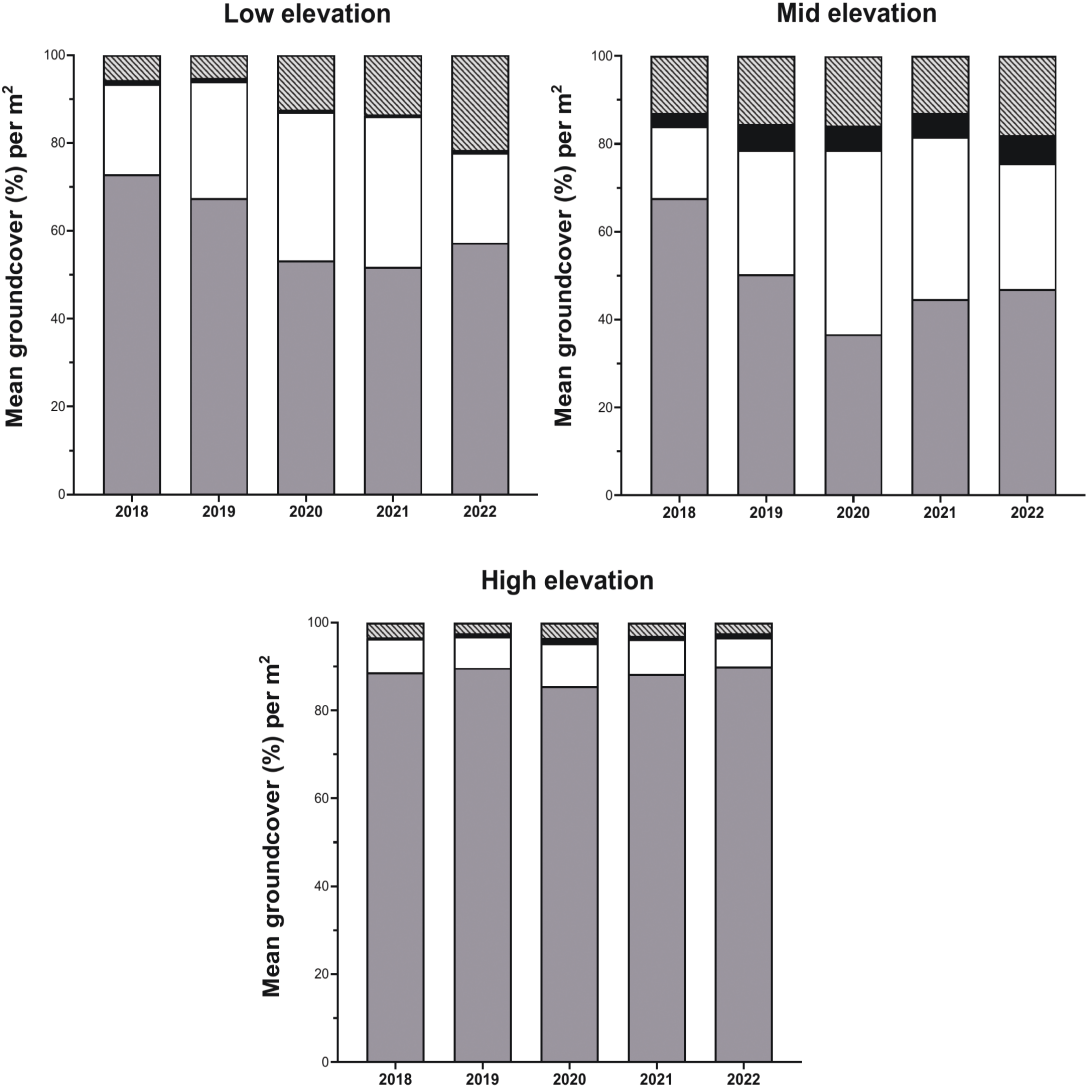
Annual variation in proportion of mean groundcover in low (500–999 m), mid (1,000–1,300 m), and high elevation (> 1,300 m) groups from 2018 to 2022 across 4 ranches in North and South Kona districts, west Hawaiʻi Island. From the bottom section of the bars to the top, grass cover shown in dark gray, forb cover in white, shrub cover in black, and bare ground in light gray stripes.

The greatest reduction in mean grass cover was observed at mid elevations (1,000–1,300 m), which decreased by 30% from 2018 to 2022 (Fig. 5). At mid elevations, grass was replaced by forbs (76% increase), bare ground (39% increase), and shrubs (7% increase) (Fig. 5). At low elevations (500–999 m), grass cover decreased by 22% from 2018 to 2022 and was replaced by bare ground (277% increase) and forbs (1% increase) (Fig. 5). In contrast, grass cover at high elevations (>1,300 m) increased by 1% (Fig. 5). Without a substantial reduction in grass cover in the high-elevations, forbs, and bare ground decreased (15% and 28% decrease, respectively) (Fig. 5).

**Fig. 6.**
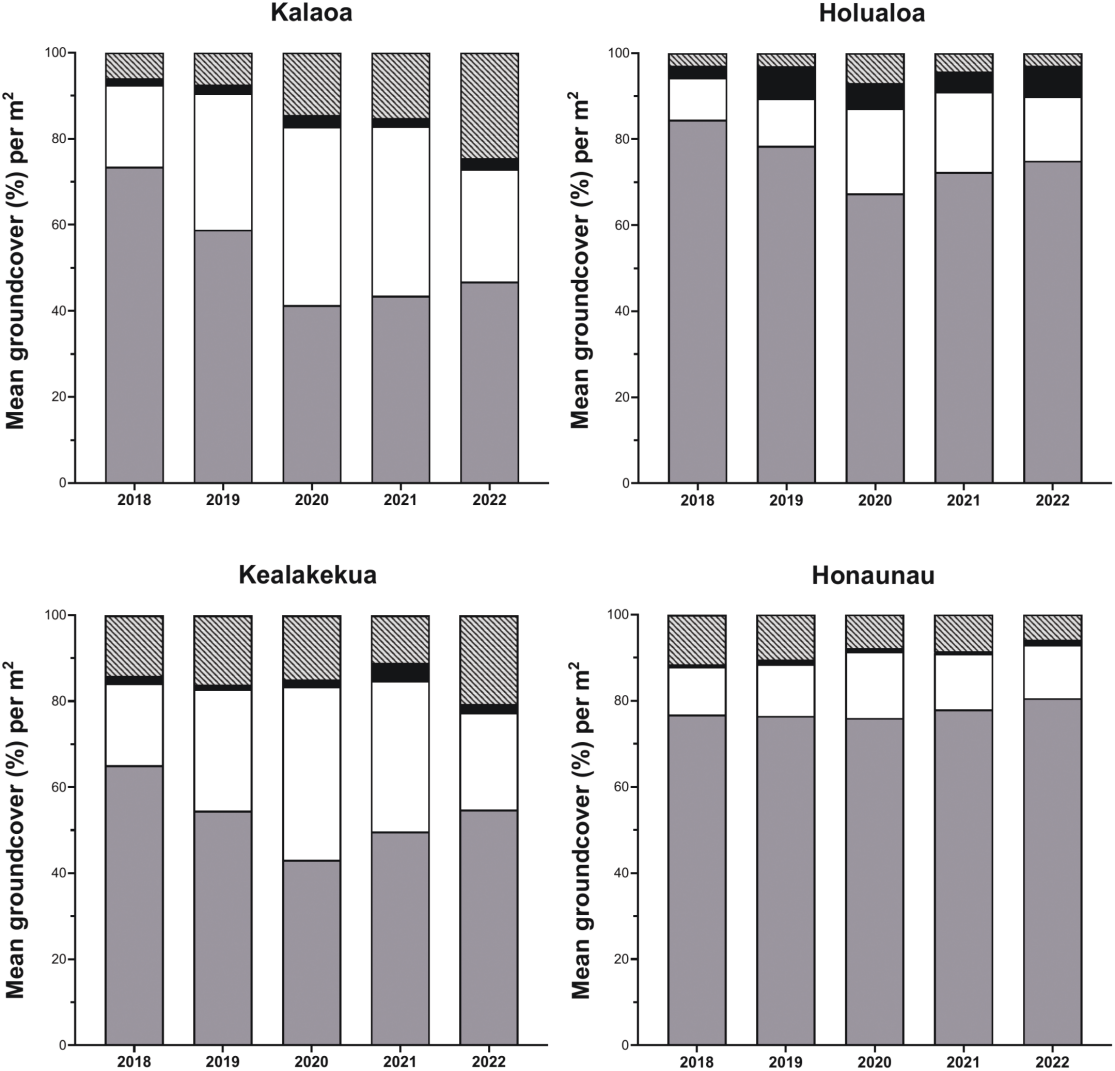
Annual variation in proportion of mean groundcover for the 4 ranches studied from 2018 to 2022 in North and South Kona districts, west Hawaiʻi Island. From the bottom section of the bars to the top, grass cover shown in dark gray, forb cover in white, shrub cover in black, and bare ground in light gray stripes.

From 2018 to 2022, mean grass cover declined at 3 of the 4 ranches: Kalaoa (36% decrease), Holualoa (11% decrease), and Kealakekua (34% decrease) (Fig. 6). Across these 3 ranches, grass was replaced by forbs (19–57% increase), bare ground (29%–303% increase), and shrubs (17%–165% increase) (Fig. 6). In contrast, grass cover increased at Honaunau by 5% and there was a significant reduction in bare ground (50% decrease), which was replaced by forbs (12% increase) and shrubs (118% increase) (Fig. 6). Across all 5 yr the highest reduction in grass cover was observed in 2020, with a 44% decrease at Kalaoa, 20% decrease at Holualoa, 34% decrease at Kealakekua, and 1% decrease at Honaunau (Fig. 6). As grass cover declined, forb cover increased by 41% at Kalaoa, 100% at Holualoa, 113% at Kealakekua, and 39% at Honaunau (Fig. 6).

## Discussion

In the present study, we conducted monthly field surveys across a broad elevational gradient on 4 west Hawaiʻi Island ranches to quantify *P. bicincta* abundance, plant associations, and changes in groundcover over time. We detected a significant effect of season and elevation on *P. bicincta* abundance. In general, *P. bicincta* nymph and adult abundance peaked during the wet season months (April–October) and at mid (1,000–1,300 m) to low elevations (500–999 m). Mean grass cover decreased the most (30%) at mid elevation sites and was replaced by forbs, bare ground, and shrubs. We detected *P. bicincta* nymphs on 32 plants including grasses, legumes, sedges, and forbs; we report 20 new *P. bicincta* plant associations.

### Nymph and Adult Abundance Patterns

Nymph abundance peaked ~1 month after the return of the wet season rains, followed by a second peak ~3 months later. This 3-month period between peaks in nymph abundance is likely due to the time required for development, mating, oviposition, and egg hatch. In Florida, the *P. bicincta* lifecycle from egg to egg averaged 76 days (Fagan and Kuitert 1969). Eggs hatched in an average of 19 days and the duration of the nymphal stage was an average of 50 days (Fagan and Kuitert 1969). Adults lived for an average of 21 days and began ovipositing after 7 days (Fagan and Kuitert 1969). The duration of each life stage varies depending on temperature and precipitation. Under optimal conditions the entire lifecycle duration plus the time needed for the next generation of nymphs to hatch is ~95 days. The interval between the 2 peaks in nymph abundance observed in this study is explained by the lifecycle duration and early instar nymph inconspicuousness (detectability) in our field surveys.

Adult abundance peaked about 2 months after the first peak in nymph abundance, reflecting their emergence from the spittle mass after completing nymph development. This was followed by a second peak in adult activity about 2 months before the onset of the next dry season. This second peak in adult abundance occurred 2 months after the first adult peak and 1 month after the second nymph peak. Site-specific climate and habitat (i.e., elevation, availability of host species, vegetative cover, soil moisture, and litter accumulation) conditions can influence diapause termination, the rate of development, and/or oviposition sites (Peck 1999, Antonatos et al. 2021, Bodino et al. 2021, Espitia Buitrago et al. 2022). These factors may have contributed to differences in population density across sites and caused a degree of variability around the population peaks for each generation (Peck 1999). After the second peak in adult abundance right before the dry season, little to no adult *P. bicincta* activity was detected until the following wet season at our sites.

The distinct abundance peaks and synchronous activity indicate 2 generations of *P. bicincta* per year in Kona. The rapid and highly synchronous outbreak of nymphs with the arrival of the rain suggests that *P. bicincta* eggs may enter diapause prior to the dry season followed by a period of postdiapause quiescence (Pires et al. 2000, Sujii et al. 2001, CIAT 2002). This is because diapausing eggs cannot respond directly to wet conditions, but the termination of diapause can be accelerated when eggs are exposed to unfavorable drought conditions and enter a state of postdiapause quiescence (Pires et al. 2000, Sujii et al. 2001, CIAT 2002). In this stage of postdiapause quiescence, eggs can respond under humid conditions and stimulate immediate eclosion, resulting in an abrupt synchronous first population peak (Pires et al. 2000, Sujii et al. 2001, CIAT 2002, Peck 2002). Conversely, when the dry season is shorter with more episodic rains before the wet season, initial outbreaks are asynchronous and less intense (Sujii et al. 2001, CIAT 2002, Peck 2002).

Long-term temporal and spatial occurrence of *P. bicincta* varied in Kona. Rainfall varies across the island and this variability becomes even more extreme with drought conditions (Luo et al. 2024). The temporal differences in abundance patterns of *P. bicincta* observed across years in this study were likely influenced by the variation in rainfall from year-to-year and the onset of drought conditions in Kona that started in late 2020 and persisted in the years following. The variability in microclimates over short distances and abrupt changes in elevation across Kona ranches may have impacted the observed spatial distribution and abundance of *P. bicincta* by influencing habitat suitability and host plant availability. Drought effects likely did not manifest evenly across Kona, so differences in plant community responses across sites may have contributed to habitat suitability for *P. bicincta* over time, ultimately impacting their distribution and abundance. The high numbers of *P. bicincta* detected in 2018–2020 in the mid elevation zone, and at 3 of the 4 ranches is likely related to the suitability of the climate conditions, pasture habitat, and host plants in these areas. Site-specific climate data may enhance our understanding of *P. bicincta* population dynamics across different microclimates in Kona. Weather stations distributed across pastures at different elevations should be utilized in future studies to obtain seasonal and annual microclimate data. Weather data can aid in the development of landscape models to predict ecological pest patterns over time and space to support appropriate management decisions (Hernández et al. 2021, Neta et al. 2023).

### Implications for Management

Understanding the timing of nymph emergence and magnitude of activity in the Kona region is crucial for developing and implementing management plans. Knowledge of these patterns can help predict the synchrony and timing of first-generation outbreaks which improves the effectiveness of management practices as it indicates the most appropriate window of time to implement control measures (Sujii et al. 2001, CIAT 2002, Peck 2002). Targeting critical periods of the lifecycle (i.e., eggs and nymphs) by creating unfavorable temperature and humidity conditions can help prevent the pest population from reaching economically catastrophic levels. Ranchers can implement grazing practices to reduce the thatch layer and accumulation of litter to expose and dry out eggs and nymphs (Wilson et al. 2023). Based on the survey data, timing of management tactics may be most effective when implemented during nymph development to reduce the population, remove the opportunity for adults to oviposit, and subject eggs at the soil surface to adverse conditions. Targeting the first generation of nymphs in the spring and early summer disrupts the lifecycle and helps reduce overall nymph density to prevent a portion of the population from reaching the highly destructive adult stage (Hewitt 1988, Wilson et al. 2023). Reducing the nymph population with management tactics aimed at the last generation of nymphs in late summer may help deter oviposition and expose overwintering eggs before the dry season returns (Hewitt 1988, Wilson et al. 2023).

### Changes in Groundcover and Plant Associations

As *P. bicincta* abundance increased at our transect sites from 2018 to 2020, grass cover decreased in response to *P. bicincta* feeding damage. The dieback of grasses shifted the dynamics of the pasture plant communities, increasing the amount of bare ground and allowing establishment of forbs and shrubs. As grasses died, legumes and forbs became more abundant and changed the availability of host plants for the next generation of *P. bicincta* nymphs.

The widespread introduction of African grasses throughout the New World tropics and subtropics has coincided with the rise in pest status of grass-feeding spittlebugs (Enkerlin and Morales 1979, Peck 1999, Holmann and Peck 2002, Thompson 2004). With no co-evolutionary history between introduced African grasses and New World Cercopidae, these grasses have not evolved defense mechanisms to avoid or tolerate spittlebug feeding (Thompson 2004, Paladini et al. 2018). The primary forage grass in Hawaiʻi is Kikuyu grass, a nitrogen-fixing (Thompson 2004) C_4_ grass of African origin, which appears to be a preferred host of *P. bicincta*. Similarly, other *Prosapia* prefer associative nitrogen-fixing introduced C_4_ grass hosts (Thompson 2004) in the New World tropics.

*Prosapia bicincta* is polyphagous and has previously been recorded on a broad range of grasses as well as hollies (*Ilex* spp., Aquifoliaceae) (Peck 1998b, Thompson and Carvalho 2016). As expected, *P. bicincta* nymphs were detected on several grasses in this study, but they were also found on sedges, legumes, and forbs; however, we did not confirm if they were able to survive on these different hosts. Due to the sampling intensity, it is possible there were errors in determining which plants the spittle masses were on depending on the spittle mass size, level of experience digging for nymphs within the spittle, and stand structure (i.e., understory vegetation density, canopy height, and density) since spittle masses often appeared to be in contact with more than one plant. *Prosapia bicincta* nymphs and adults are opportunistic xylem feeders, so as early instar nymphs with limited mobility, they may initially feed on the roots of plants in their proximity until they develop further and can move to distant locations to find a more suitable grass host or overcome plant architectural barriers restricting early instars (i.e., tissue hardness, presence of trichomes, nymph stylet length vs. trichome height, xylem tension and depth, concentration of amino acids, etc.) (Pass and Reed 1965, Fagan and Kuitert 1969, Hoffman 1983, Espitia Buitrago et al. 2022). Nymphs cannot produce spittle without feeding (Thompson et al. 2023), so they may have used these plants as xylem sources for transitional spittle masses rather than using them to complete their life cycle. Observations of *P. bicincta* on new or unexpected plants may be the result of transient associations rather than viable host associations.

Damaged pastures experienced landscape-level changes as shown by the changes in groundcover. When grasses died from the damage inflicted by previous *P. bicincta* generations they were replaced by legumes and forbs, which may have forced a change in nymphal food plants. The widespread incursion of forbs like fireweed (*Senecio madagascariensis* Poir.) and marestail (*Erigeron canadensis* L.) into damaged pastures may have provided nymphs with new host choices. When their preferred associative nitrogen-fixing C_4_ grass hosts die off, it is possible *P. bicincta* resort to feeding on nitrogen-fixing legumes, such as the 4 legume species we reported as new food plants of *P. bicincta*.

The changes in groundcover reported in the present study suggest that *P. bicincta* infestations have caused widespread and long-term damage to pastures of Kikuyu and pangola grass in the North and South Kona districts, ultimately resulting in landscape transformation through invasion and establishment of invasive weeds and low-quality forage grasses (Thorne et al. 2018, Wilson et al. 2023). Damage caused by the invasion of *P. bicincta* will necessitate a shift in the conventional rangeland management practices. *Prosapia bicincta* management in pastures should be developed based on site-specific information due to the variation in elevation, climate conditions, and plant communities across the Hawaiʻi Island landscape (Peck 1998a, Valério et al. 2001).

The pattern of first detection of *P. bicincta* in 2016, to a population peak from 2018 to 2020, and then a population decline from 2021 to 2022 is analogous to patterns observed in the Emerald ash borer *Agrilus planipennis*, across eastern North America (Rutledge and Clark 2023). After invasion into new areas, *A. planipennis* infests all available ash trees and experiences a period of rapid population growth (Garnas et al. 2023, Rutledge and Clark 2023). Once the population peaks, the available ash trees die off, causing the population to crash (Garnas et al. 2023, Rutledge and Clark 2023). Thus, patterns of *A. planipennis* population dynamics are primarily driven by the availability of ash trees. After 3 yr, *P. bicincta* may have contributed to its own population decline by depleting host plant resources and causing long-term changes to rangeland plant communities that resulted in unfavorable habitat. Continued monitoring at these sites and the surrounding areas are needed to determine if this population decline is permanent or varies over longer time scales.

## Conclusion

This is the first study to quantify abundance and plant associations for *P. bicincta* in Hawaiʻi and estimate its impacts on vegetative cover in Kona pastures. Five years of field surveys revealed wet season months (April–October) consistently had significantly higher *P. bicincta* abundance relative to dry season months (November–March) and most activity was detected at mid elevation pastures located between 1,000 and 1,300 m a.s.l. We also observed a steady increase in abundance during the first 3 yr of surveys, followed by a decline in populations perhaps associated with host plant availability, local variability in climate conditions, and/or dispersal behavior. Grasses accounted for 72% of the plants that *P. bicincta* nymphs were detected on. Pastures located between 1,000 and 1,300 m a.s.l. in elevation, where *P. bicincta* activity was highest, had the biggest decrease in mean grass cover and greatest increase in forb and shrub cover. Areas with high *P. bicincta* infestations experienced severe dieback of pasture grasses used for livestock forage, allowing for the establishment of weedy forbs and shrubs. This study provides a basis for estimating *P. bicincta* developmental rates, which can be used to determine optimal timing of management practices and target vulnerable stages of the lifecycle.

## Supplementary Material

nvae062_suppl_Supplementary_Material
