# Supplementary material for: Prosapia bicincta (Hemiptera: Cercopidae) abundance, plant associations, and impacts on groundcover in Hawaiʻi Island rangelands
Source: Environ Entomol. 2024 Jul 2;53(5):870–80. doi: 10.1093/ee/nvae062 (PMC13011921; doi:10.1093/ee/nvae062)
Supplement: nvae062_suppl_Supplementary_Material [file nvae062_suppl_supplementary_material.pdf]

# Supplemental Figures with Legends

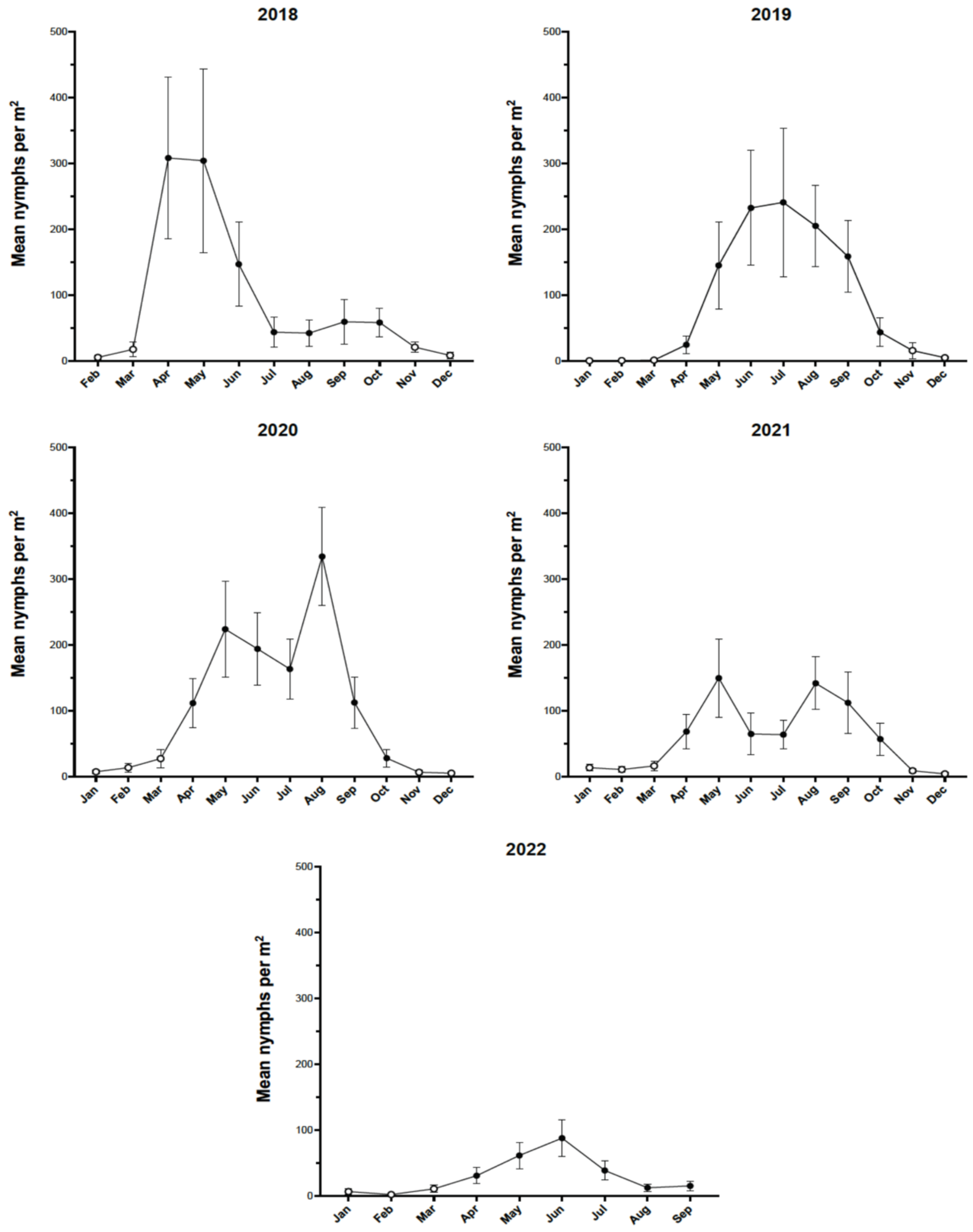


**Supp. Figure S1.** Annual variation in *Prosapia bicincta* nymph abundance (mean ± SEM) from 2018-2022 in North and South Kona districts, west Hawai‘i Island.

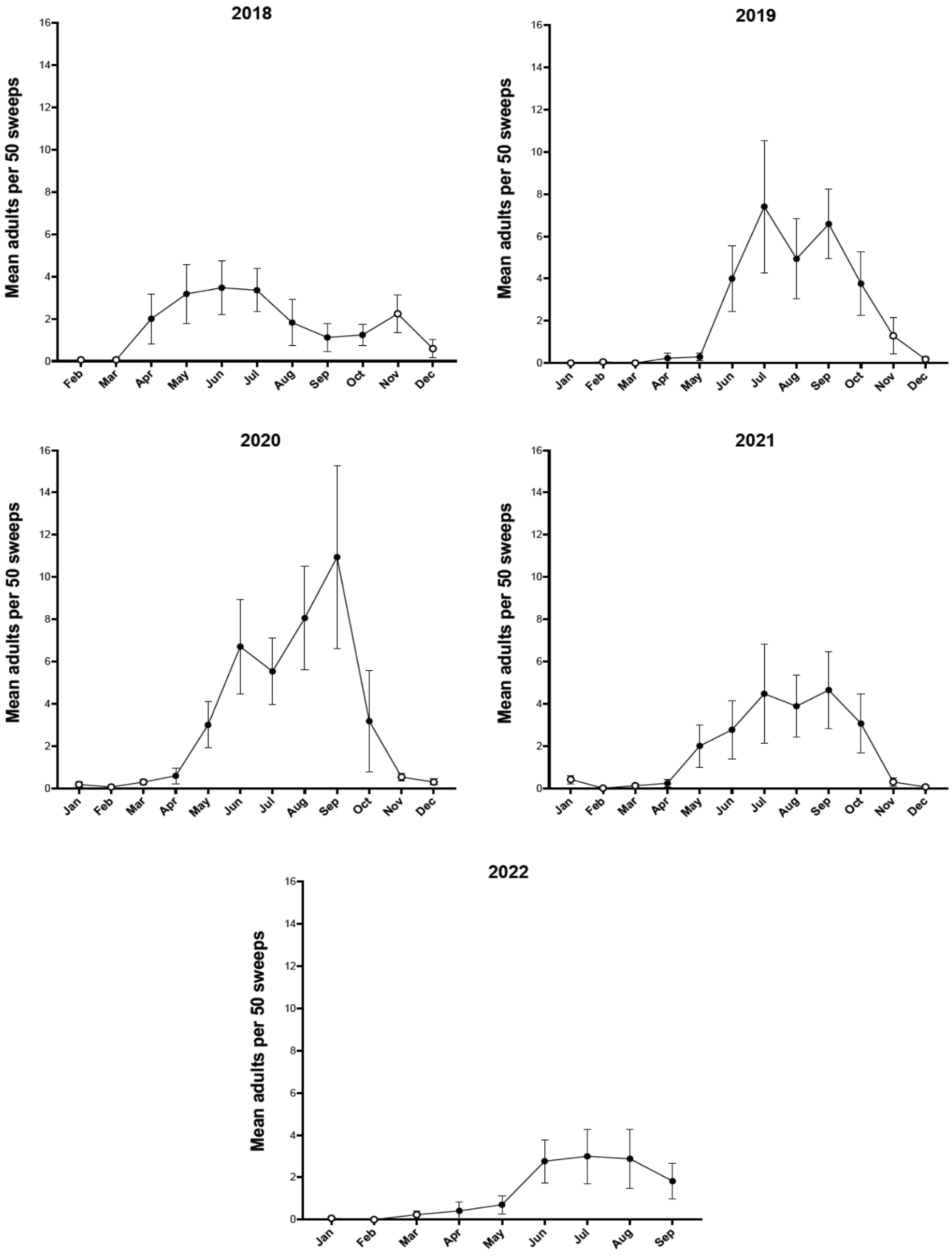


**Supp. Figure S2.** Annual variation in *Prosapia bicincta* adult abundance (mean ± SEM) from 2018-2022 in North and South Kona districts, west Hawai'i Island.

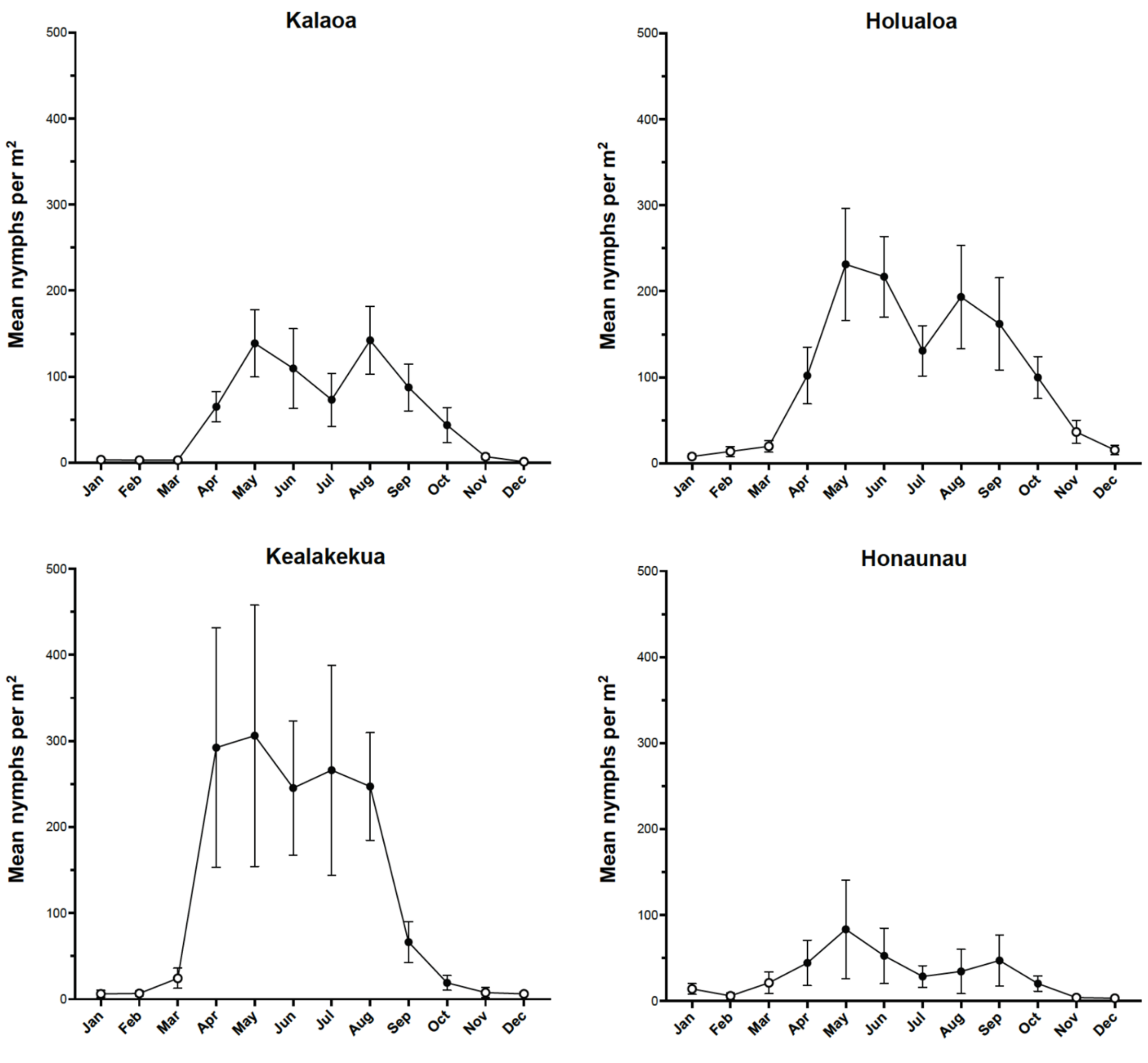


**Supp. Figure S3.** Seasonal variation in *Prosapia bicincta* nymph abundance (mean ± SEM) across four ranches located in North and South Kona districts, west Hawai'i Island. Elevations of the sampling areas ranged from 736-1,049 m at Kalaoa, 1,072-1,605 m at Holualoa, 519-1,194 m at Kealakekua, and 1,102-1,874 m at Honaunau.

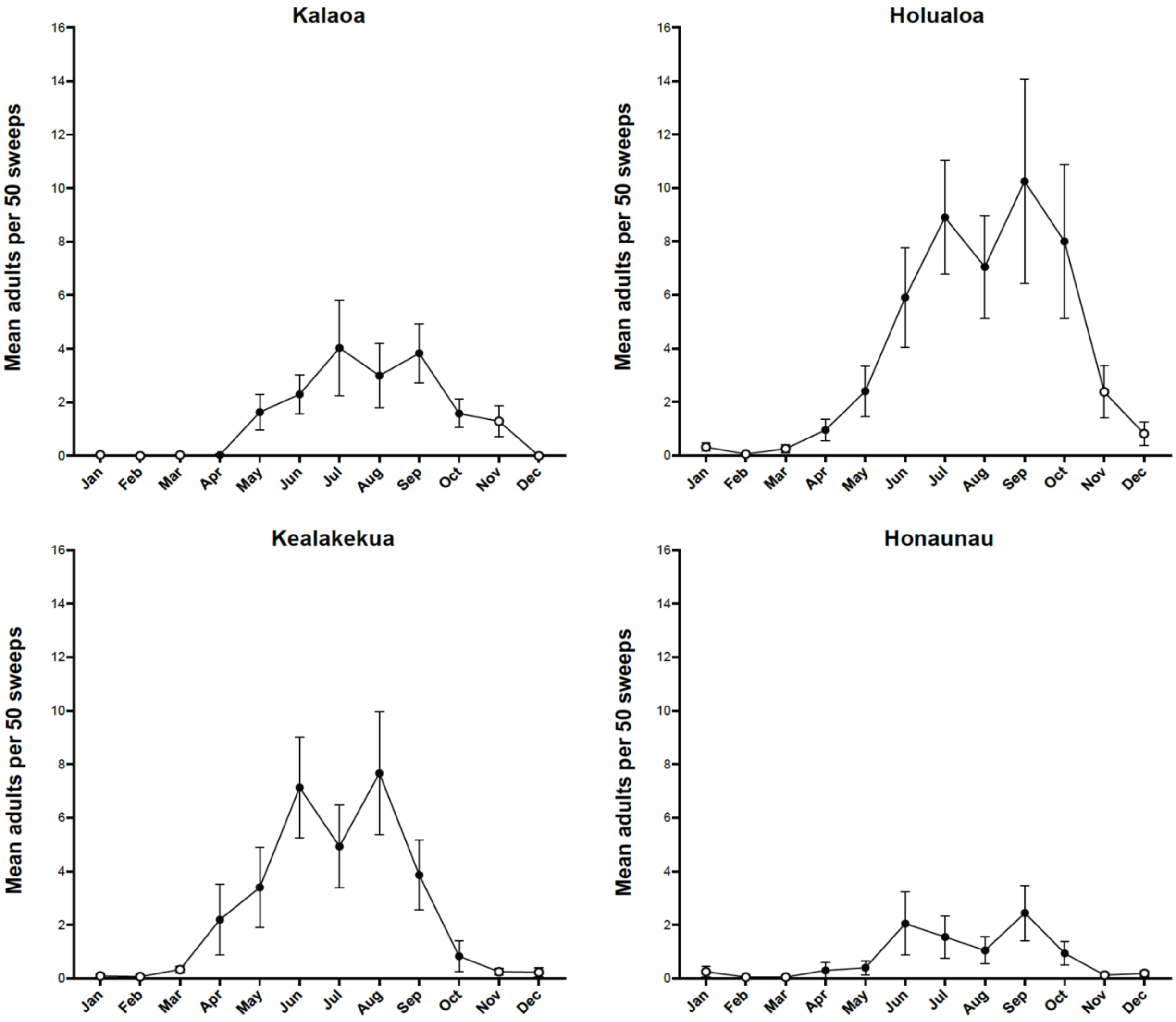


**Supp. Figure S4.** Seasonal variation in *Prosapia bicincta* adult abundance (mean ± SEM) at four ranches located in North and South Kona districts, west Hawai'i Island. Elevations of the sampling areas ranged from 736-1,049 m at Kalaoa, 1,072-1,605 m at Holualoa, 519-1,194 m at Kealakekua, and 1,102-1,874 m at Honaunau.

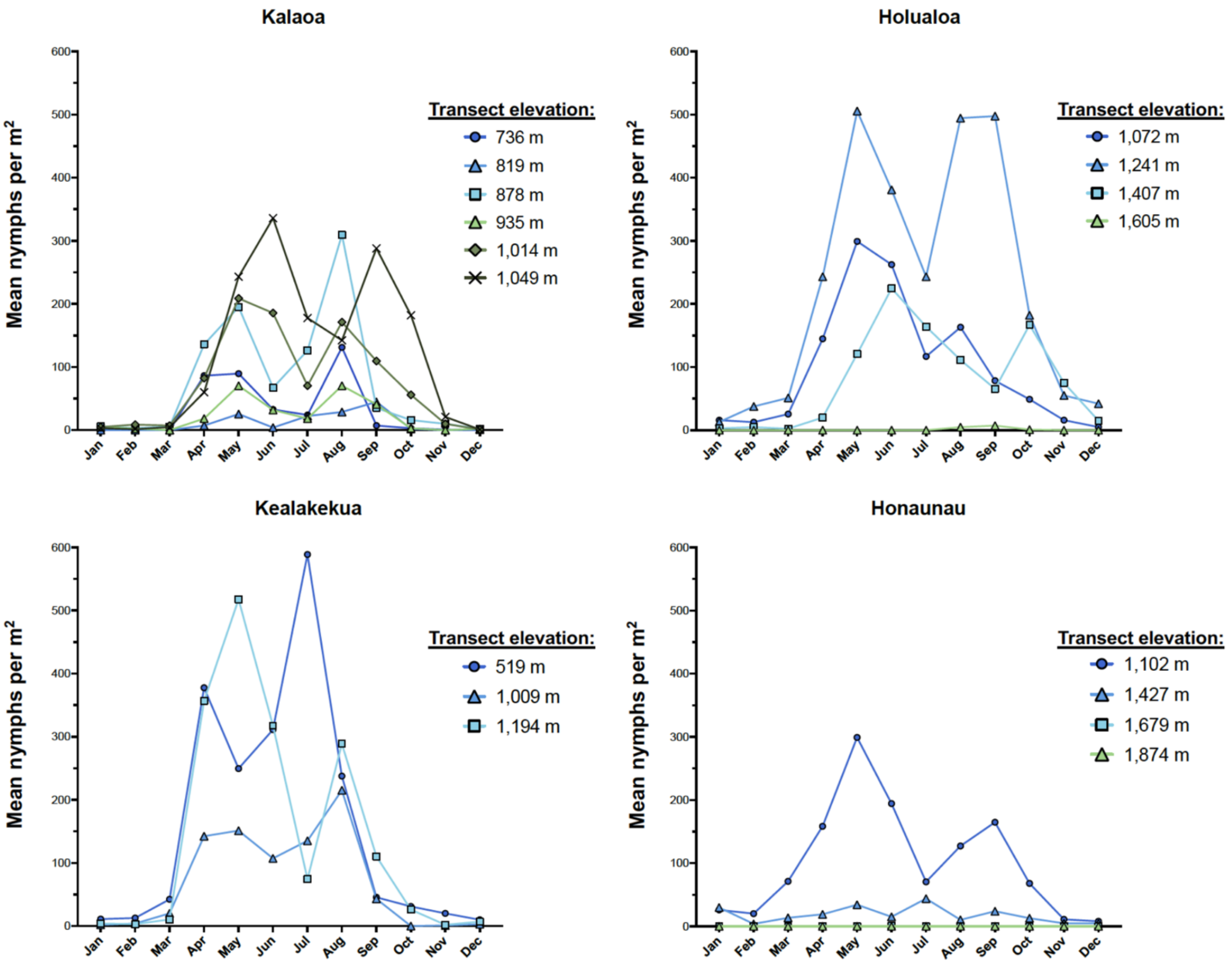


**Supp. Figure S5.** Seasonal and geographic variation in *Prosapia bicincta* nymph abundance (mean) from 2018-2022 at four ranches in North and South Kona districts, west Hawai'i Island. The mean number of nymphs is shown for each elevation sampled per ranch. The number of transects at each ranch varied and not all locations had the same range of elevations. Elevations were dictated by transect locations in suitable habitat.

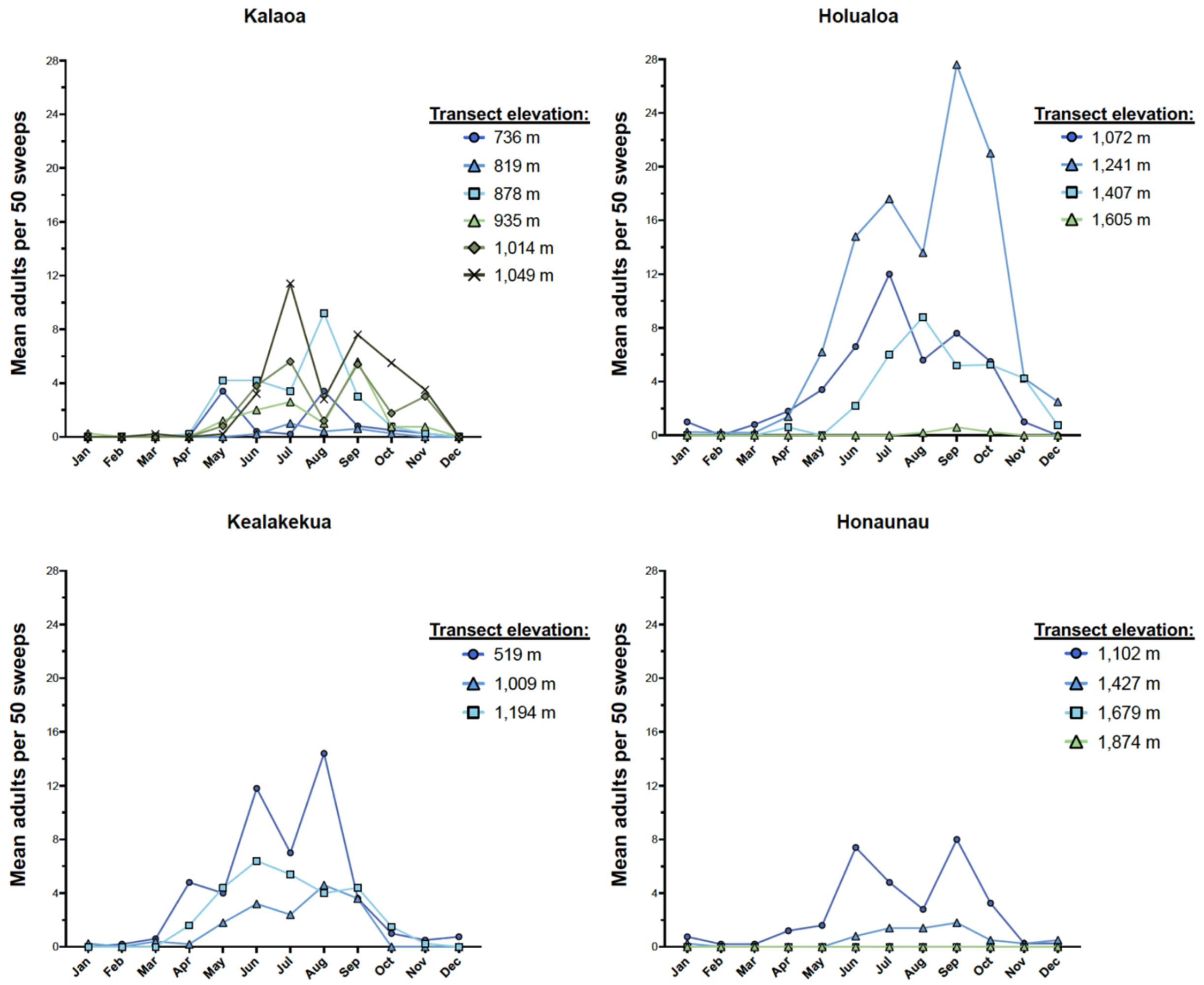


**Supp. Figure S6.** Seasonal and geographic variation in *Prosapia bicincta* adult abundance (mean) from 2018-2022 at four ranches in North and South Kona districts, west Hawai‘i Island. The mean number of adults is shown for each elevation sampled per ranch.
